# What are innovations in peer review and editorial assessment for?

**DOI:** 10.1186/s13059-020-02004-4

**Published:** 2020-05-04

**Authors:** Willem Halffman, Serge P.J.M Horbach

**Affiliations:** Institute for Science in Society, Radboud University Nijmegen, Nijmegen, The Netherlands

Peer review at research journals is going through a period of intense innovation. Some journals are experimenting with ‘open’ review procedures that reveal identities or even review reports; some with pre-registered reports that shift review attention to experimental protocols rather than to focus on results; or with post-publication review through readership commentary [1]. Well-resourced journals embed peer review in editorial review procedures that may include text similarity scanners, language, or reference checks, or that involve low-wage sub-contracting of editorial work in highly distributed procedures. With increasing IT support and editorial division of labour, peer review is but one link in the chain that guards, selects and improves manuscript quality, as part of editorial procedures that are now more diverse than ever—even though the majority of research journals still uses fairly standard peer review procedures and more radical innovations are limited to a few research niches [2]. How can we learn from all these innovations?

## Diversity in expectations

An important driver of current editorial innovations is a set of diverse and occasionally incongruous expectations. Perhaps most telling in this respect is the question of whether peer review is just meant to distinguish correct from incorrect research or whether it should also distinguish interesting and relevant from less important or even trivial research. High-volume journals such as the *PLoS* series ask their reviewers to merely assess whether reported results are correct, not whether they are novel or earth-shattering. As a result, these journals publish very large numbers of open access articles, with relatively moderate Author Processing Charges. On the other end of the spectrum, journals like *Nature* or *Science* will not publish even the most solid research without important news value for their wide and interdisciplinary readership. Should peer review distinguish between important and less important findings? The grounds on which peer review and wider editorial assessment are to select papers for publication are closely related to journal business models.

The diversity of expectations for peer review is even bigger if we consider the variation between research fields. It is easy to slip into the research equivalent of ethnocentrism: to think that all research fields basically work like our own—or would be better off if they did. The editorial assessment of experimental genetics is quite a different matter from the assessment of a climate model, a mathematical proof, a geological measurement, or even further afield: qualitative social science. The scholarly publication system caters for a wide range of research endeavours. The growing diversity of publication practices and the specific ways in which these assess the value of contributions should come as no surprise.

## Replication and misconduct

Other concerns driving peer review innovations have included the ‘replication crisis’: the worry that many published results appear hard to replicate and that this endangers the very core of the scientific endeavour [3]. Improved peer review and improved editorial procedures in which peer review is embedded are also seen as a way to make sure that what gets published is also truly reliable.

Unreproducible research may not necessarily be wrong, but simply incompletely reported. Hence, various initiatives have been developed to increase the detail in research reports, in particular with respect to methods. These include checklists for biomedical research materials [4], for the adequacy of animal research reports [5], instructions to improve materials’ identification [6], or to improve research materials’ validation [7]. Such initiatives may provide extra information allowing peer reviewers and readers to verify reported results, but may also act as nudges to authors, or as publication checks used directly by editorial staff (rather than peer reviewers).

Instead of relying entirely on the personal expertise of reviewers, checklists and publication guidelines aim to improve the scientific record through proceduralisation: researchers are expected to improve the reproducibility or even reliability of their work by having to provide detailed methodological information. For example, methodological publication guidelines may not only encourage researchers to more adequately report the identity of research animals, antibodies, or cell lines. Some concerned commentators also hope this will actually raise the standards of animal testing (such as through randomisation or blinding), improve the validation of antibodies, or eradicate the festering problem of misidentified cell lines [8].

Even more alarming reasons for editorial innovations have been based on worries over research fraud. While it can be argued that peer reviewers or even editors cannot be held accountable for malicious practices of their authors, checks for plagiarism, duplicate publications, statistical data manipulation, or image doctoring do suggest at least some responsibility is expected from and taken by journals. This responsibility extends to clear and forthright action after problematic publications have been discovered, such as through retractions, the large majority of which involve misconduct [9]. While the expectations may be high for editors to take action against fraud, from retracting papers to warning authorities or host institutions, this may also put a considerable additional burden on editorial offices. This is especially the case since misconduct may not always be clear-cut and allegations may be challenged by the accused, who are also entitled to fair treatment and protection from slander.

Editorial innovations in response to replication and misconduct concerns are also stimulated by the affordances of information technology or shifts in publication business models. On the affordance side, electronic publishing and booming data science resources have facilitated the development of text similarity scans, with an expansion from applications in the policing of student plagiarism to scientific publishing. In a similar vein, semi-automatic statistics scanners and tools to flag falsified or copied images are now in development. Here too, commercial considerations play a role. Advertised as a way to improve the quality of published research, scientific publishers can also deploy such technology-supported editorial checks as justifications for relatively costly publishing formats, in the face of looming community-managed open access initiatives ranging from pre-print servers to meta-commentary initiatives such as PubPeer.

## Unclear efficacy

Much as innovations in editorial procedures are advocated by scientists and publishers on a mission to raise research literature standards, the evidence for the efficacy of these innovations is patchy and sometimes even contradictory. Some of the innovations move in opposite directions: increasing objectivity of reviews can be presented as a reason for increased anonymity, but also for revealing identities of all involved. ‘Double blind’ reviews (or even ‘triple blind’, if author and reviewer identities are anonymised to editors) are expected to encourage reviewers and editors to focus on content, rather than to be influenced by authors’ identities, affiliations, or academic power positions. Inversely, revealing identities, or even publishing review reports, can also be presented as beneficial: as a form of social control making reviewers accountable, in which it is not possible to hide improper reviews behind anonymity, or in which the wider research community can keep a vigilant eye. The key question in the blindness-versus-openness debate has been what constitutes the best way to neutralise bias or unfairness based on personal dislike, power abuse, disproportionate respect for/abuse of authority, rudeness, gender, institutional address, or other social processes that editorial fairness is expected to neutralise. So far, no conclusive evidence has been presented for the superiority of either strategy.

A similar shortage of evidence is witnessed in the case of journals’ methodological guidelines and reporting standards. While guidelines and checklists may improve the identification of research materials in published papers, guidelines do not work by themselves. Guidelines require active implementation by journals and some degree of support from the research community on which journals rely for the continued submission of manuscripts. For example, journals cannot police scientific rigour beyond what their research constituency as a whole is willing to provide. In the face of publication pressures or the costs of extra validation testing, improved reporting seems to focus on more easily fixable *identification* rather than deeper *validation* of research materials. Furthermore, if researchers provide antibody validation information, this also requires expertise on validation procedures among reviewers or editors, which may not be obvious in all fields using antibodies as research tools. (For similar reasons, some journals now work with statisticians as part of a growing specialisation in review to cover specific methodological issues.) Such guidelines need to be well-embedded and enforced if they are to fundamentally improve methodological procedures.

## The publishing landscape

The vivid diversity and innovation in editorial policies creates exciting opportunities to learn from each other. The use of checklists and other reviewer instructions, specialisation of reviewers, post-publication review and correction practices, and similar innovations may well be of far wider use than the journals that are currently experimenting with them. One condition for learning is that editorial assessment is visible and transparent [10]. It is quite puzzling to see how many journals still simply announce that they ‘use peer review to assess papers’, as if that explains how papers are handled. Another condition is that innovation processes have to respect the diversity of research cultures. For example, large publishers, catering for a wide range of research fields, are well aware that one size does not fit all: there is not one best way to organise editorial assessment, but this should not preclude possibilities to try out innovations that seem to work well elsewhere.

More systematic evaluation of how innovations change editorial assessment would certainly also help this learning process. However, given the wide range of expectations and motivations involved, evaluating the effects of editorial innovations is complex. For example, whether single or double blind is ‘better’ is not just a matter of whether more errors are filtered out, but also of fairness (gender, institutional address), of whether the more significant papers are (or should be) selected, whether reproducibility is improved, whether fraud is traced, and all these other mixed or even incompatible expectations.

Moreover, the possibilities for editorial improvement do not present themselves in a void. Reasonable if complex arguments have to be measured against systemic realities of the research world. A prominent factor here is publishing economics. After a wave of concentration in the research publishing industry [11], the large publishers are now developing strategies to survive and thrive in the age of ‘open science’. While science policy is pushing for open data and open access publishing, some publishers aim to develop new business models based on indicators, databases, and similar uses of meta-data in search engines and research assessment tools. Their willingness to adopt editorial innovations depends on their strategic choices and business models, which seem increasingly focused on turnover, efficiency, and advanced division of labour in highly structured and automated publication management systems.

Another context that conditions our options for innovation is the research evaluation system: how we assess scientific achievements, award career advancement, or distribute resources between research institutes and teams. Unfortunately, the development of publication-based indicators (such as publication counts, citation counts, h-factors, or impact factors) has pushed the research publication system to its limits. Many researchers now submit papers ‘to get a publication’, spurred on by tenure-track criteria, competitive job pressure, and sometimes even considerable financial bonuses—and quite understandably so, as their careers as scientists may depend on it. Young researchers need to ‘score’ with prominent publications, and our journals need to cater for this too, at least for the time being. While the obsession with ‘output measurement’ has spread from the Anglo-Saxon world to emerging research cultures such as China, where it has now taken perhaps its most extreme form [12], even metrics developers are coming to their senses and are advocating research evaluation that returns to ‘quality over quantity’ [13], but this will take time.

Reflecting on a future of careful editorial assessment and meaningful peer review therefore also requires us to pause and think about what is at stake in how we share our research findings. Do we always need the high-speed production of factoids, the citation-scoring career-boosting mediated-but-hastily-published papers that end up needing corrections further down the line? Or is there something to be said for slowing down, in a research world that aims more at cooperative advancement of knowledge rather than ‘scoring’? The daily practice of how we run and try to improve our journals reflects these big questions as much as the small, technical ones.
